# Targeting of retrovirus-derived *Rtl8a*/*8b* causes late-onset obesity, reduced social response and increased apathy-like behaviour

**DOI:** 10.1098/rsob.240279

**Published:** 2025-01-29

**Authors:** Yoshifumi Fujioka, Hirosuke Shiura, Masayuki Ishii, Ryuichi Ono, Tsutomu Endo, Hiroshi Kiyonari, Yoshikazu Hirate, Hikaru Ito, Masami Kanai-Azuma, Takashi Kohda, Tomoko Kaneko-Ishino, Fumitoshi Ishino

**Affiliations:** ^1^ Department of Epigenetics, Medical Research Institute (MRI), Tokyo Medical and Dental University (TMDU), Tokyo 113-8510, Japan; ^2^ Center for Experimental Animals, TMDU, Tokyo 113-8510, Japan; ^3^ Faculty of Life and Environmental Sciences, University of Yamanashi, Kohfu, Yamanashi 400-8510, Japan; ^4^ Division of Cellular and Molecular Toxicology, Center for Biological Safety and Research, National Institute of Health Sciences (NIHS), Kawasaki, Kanagawa 210-9501, Japan; ^5^ Laboratory for Animal Resources and Genetic Engineering, RIKEN Center for Biosystems Dynamics Research, Kobe, Hyogo 650-0047, Japan; ^6^ Research Facility Center for Science and Technology, Kagawa University, Takamatsu, Kagawa 761-0793, Japan; ^7^ Faculty of Nursing, Tokai University School of Medicine, Isehara, Kanagawa 259-1193, Japan

**Keywords:** retrovirus-derived genes, neuronal development, social response, apathy, late-onset obesity, Prader–Willi syndrome

## Abstract

Retrotransposon Gag-like (RTL) 8A, 8B and 8C are eutherian-specific genes derived from a certain retrovirus. They cluster as a triplet of genes on the X chromosome, but their function remains unknown. Here, we demonstrate that *Rtl8a* and *Rtl8b* play important roles in the brain: their double knockout (DKO) mice not only exhibit reduced social responses and increased apathy-like behaviour, but also become obese from young adulthood, similar to patients with late Prader–Willi syndrome (PWS), a neurodevelopmental genomic imprinting disorder. Mouse RTL8A/8B proteins are expressed in the prefrontal cortex and hypothalamus and localize to both the nucleus and cytoplasm of neurons, presumably due to the N-terminal nuclear localization signal-like sequence at the N-terminus. An RNAseq study in the cerebral cortex revealed reduced expression of several GABA type A receptor subunit genes in DKO, in particular *Gabrb2,* which encodes its β2 subunit. We confirmed the reduction of GABRB2 protein in the DKO cerebral cortex by western blotting. As GABRB2 has been implicated in the aetiology of several neurodevelopmental and neuropsychiatric disorders, it is likely that the reduction of GABRB2 is one of the major causes of the neuropsychiatric defects in the DKO mice.

**Figure:**
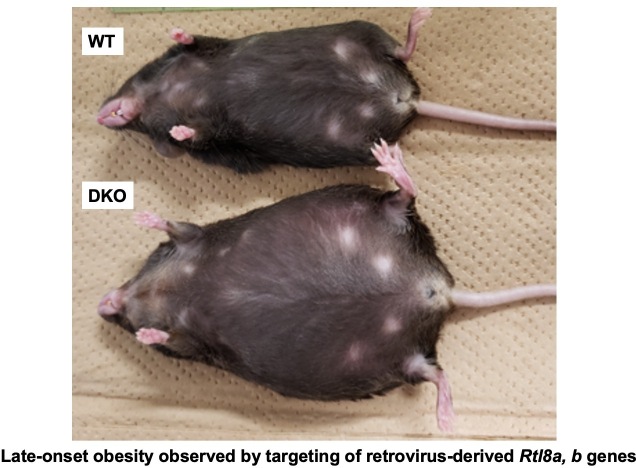

## 1. Introduction

In the human genome, there are 11 retrotransposon Gag-like (RTL*,* also known as sushi-ichi-related retrotransposon homologue (SIRH)) genes that encode proteins homologous to the sushi-ichi GAG (and sometimes also to POL) protein(s) [1–5]. Recently, it has been reported that RTL/SIRH genes were domesticated from three independent lineages of Ty3/gypsy (formally known as *Metaviridae*) [6]. Importantly, certain RTL/SIRH genes play essential and/or important roles in eutherian development [7–11], such as paternally expressed 10 (*Peg10*, aka *Sirh1* or *Rtl2*) [5,12], *Rtl1* (aka *Peg11* or *Sirh2*) [13] and leucine zipper downregulated in cancer 1 (*Ldoc1*, aka *Rtl7* or *Sirh7*) [14] in the placenta, and *Rtl4* (aka *Sirh11* or *Zcchc16*) [15,16], *Rtl1* [17,18], *Rtl6* (aka *Sirh3*) [19], *Rtl5* (aka *Sirh8*) [19] and *Rtl9* (aka *Sirh10*) [20] in neuronal function and/or innate immune system in the brain. *Rtl1* is also important for muscle development [21].

The biological roles of *RTL8A*, *8B* and *8C* (aka *SIRH5*, *6* and *4*) remain unknown, although their involvement has recently been implicated in two human diseases, Angelman syndrome (AS) [22] and amyotrophic lateral sclerosis (ALS) [23]. AS is a neurodevelopmental genomic imprinting disorder caused by paternal duplication of chromosome 15q11-q13 harbouring maternally expressed ubiquitin protein ligase E3A (*UBE3A*) or *UBE3A* mutations [24–26]. Both RTL8A-C and PEG10 proteins, another RTL gene product, were increased in differentiated neurons from AS patient iPSCs, suggesting that both RTL8A-C and PEG10 proteins are direct targets of UBE3A [22]. Therefore, it is likely that RTL8A-C is decreased in Prader–Willi syndrome (PWS) which is caused by maternal duplication of the same chromosomal region associated with a double dose of UBE3A. PWS is another neurodevelopmental genomic imprinting disorder characterized by late-onset obesity and autism spectrum disorder (ASD)-like symptoms from the juvenile period [24,27]. Whiteley *et al*. demonstrated that RTL8A-C decreased but PEG10 increased in the differentiated neurons of ALS patient iPSCs, suggesting that PEG10 is a direct target of ubiquitin-like protein (ubiquilin) 2, UBQLN2 [23], a major gene for responsible for ALS [28], and also that RTL8A-C is protected from proteasome-dependent degradation by complex formation with UBQLN2. It is also reported that RTL8 is required for the nuclear translocation of UBQLN2 and thus for quality control in the neuronal nuclei [29].

Inhibitory neurotransmission is primarily mediated by γ-aminobutyric acid (GABA), which activates synaptic GABA type A (GABA_A_) receptors. In humans, GABA_A_ receptors are composed of pentameric subunits selected from eight different classes, such as α1−6, β1−3, γ1−3, δ, ε, π, θ, ρ1−3 [30,31]. The major isoform of GABA_A_ receptors in the brain is composed of two α1, two β2 and one γ2 subunits [32,33]. Importantly, several GABA_A_ receptor subunits have been shown to be associated with psychiatric disorders. For example, alterations in the GABA_A_ receptor subunit beta2 (*GABRB2*) have been implicated in several neuropsychiatric and neurodevelopmental disorders, including schizophrenia (SZ), bipolar disorder (BP), frontotemporal dementia (FTD) and ASD [34–41]. *GABRB2* has also been reported to be associated with epilepsy, Alzheimer’s disease, substance dependence, depression, Internet gaming disorder and premenstrual dysphoric disorder [42].

In this work, we addressed the biological function of *Rtl8a* and *Rtl8b* using their double knockout (DKO) mice and demonstrated that they play an important role in neuropsychiatric behaviour, possibly via *Gabrb2* downregulation. We also discuss the possible association of RTL8 downregulation with PWS, since the phenotypes of the *Rtl8a* and *Rtl8b* DKO mice are quite similar to those of patients with late PWS, ASD-like behaviours as well as late-onset obesity.

## 2. Results

### 2.1. Conservation of *RTL8A-C* in eutherians

*RTL8A-C* (aka *SIRH5*, *6* and *4*) forms a multi-gene cluster on the X chromosome. In eutherians, its number (2–4, mostly 3, excluding pseudogenes) and amino acid (aa) sequence are well conserved (electronic supplementary material, table S1, red: dN/dS < 0.1, pink: 0.1 ≤ dN/dS < 0.2), suggesting its important role in eutherians. In most cases, *RTL8*s within the same species exhibit the highest homology to each other compared with those in other species, suggesting independent gene conversion events in each species, and therefore are not in a precise orthologous relationship among eutherians (electronic supplementary material, figure S1) [43].

**Figure 1.**
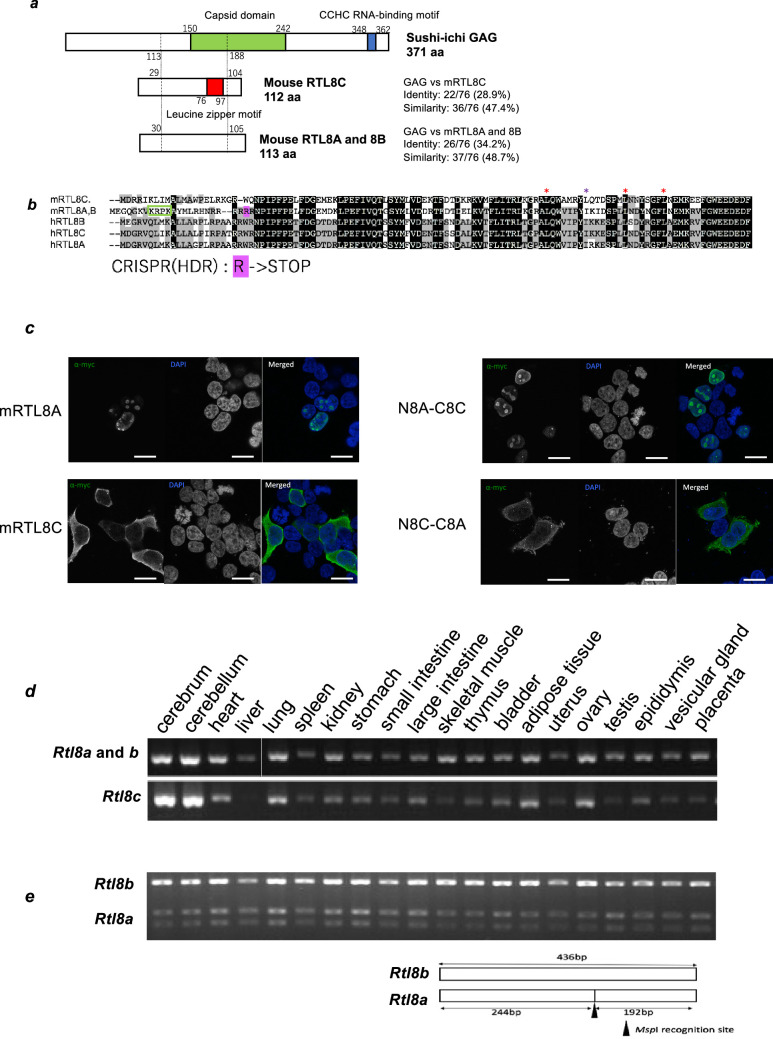
Characteristics of mouse RTL8A-C proteins. (*a*) Both mRTL8A and 8B comprise 113 aa with an identical aa sequence while mRTL8C comprises 112 aa including a unique leucine zipper motif (the red box) (see also electronic supplementary material, figure S2). (*b*) Alignment of mouse and human RTL8A-C. The leucine zipper motif of mRTL8C is indicated by three purple asterisks (top). It should be noted that the second leucine (the black asterisk) is unique to mRTL8C (see also electronic supplementary material, figure S2). mRTL8A/8B has a rodent-specific nuclear localization signal (NLS)-like peptide in the N-terminus (the green box). A stop codon shown in pink was replaced with arginine (R) at 23 aa from the N-terminus in *Rtl8a* and *8b* DKO. (*c*) Subcellular localization of mRTL8A and mRTL8C proteins (left), and chimera proteins that have the N-half of mRTL8A and C-half of mRTL8C (N8A-C8C) and the N-half of RTL8C and C-half of RTL8A (N8C-C8A). Scale bar, 20 μm. (*d*) Expression profiles of *Rtl8a/8b* and *Rtl8c.* RT-PCR results in adult tissues and organs are shown. (*e*) RFLP analysis of *Rtl8a* and *8b*. The upper bands represent *Rtl8b* expression while the bottom two bands represent *Rtl8a* expression. The black arrowhead indicates the location of the recognition site of MspI.

In mice, *Rtl8a* and *Rtl8b* encode identical proteins with 113 aa, while *Rtl8c* encodes a protein with 112 aa exhibiting 70% aa identity to the former (figure 1*a*,*b*; electronic supplementary material, figure S2). They share identity with the suchi-ichi retrotransposon GAG (48.7 and 47.4% similarity and 34.2 and 28.9% identity, respectively). Mouse RTL8A-C (mRTL8A-C) proteins share a high overall aa sequence homology with those of other eutherian species (electronic supplementary material, table S1, figure S2), but mRTL8A (also mRTL8B) has a rodent-specific nuclear localization signal (NLS)-like sequence (K-K/R-X-K/R) in the N-terminus [44], whereas mRTL8C has a unique leucine zipper motif (76–97 aa) (figure 1*a*,*b*, and electronic supplementary material, figure S2).

We examined the subcellular localization of the mRTL8A and 8C proteins in NIH3T3 cells by plasmid overexpression: the former in the nuclei (nucleoli) and the latter in the cytoplasm (figure 1*c*, left). In addition, the subcellular localization of two chimeric proteins of the N- and C-terminal halves of mRTL8A and 8C, such as N8A-C8C and N8C-C8A, was determined by their N-terminus (figure 1*c*, right), suggesting that the rodent-specific NLS-like sequence of mRTL8A (and 8B) can function *in vivo.*

*Rtl8a-c* mRNAs exhibit higher expression in the cerebrum and cerebellum, but are ubiquitously expressed in all other tissues and organs, with the exception of no *Rtl8c* expression in the liver (figure 1*d*). *Rtl8a* and *Rtl8b* exhibit similar expression patterns as estimated by a DNA polymorphism analysis using an MspI recognition site (figure 1*e*). To address their biological functions and the reason why they exist as a cluster of multiple genes, we first generated *Rtl8c* flox mice to generate TKO mice in the future, and then both *Rtl8a* and *Rtl8b* were knocked out by introducing a stop codon at a site 23 aa from the N-terminus of each gene using CRISPR/Cas9 mediated genome editing (electronic supplementary material, figures S3–S6).

### 2.2. Reproductive and growth abnormalities in *Rtl8a*/*8b* double knockout mice

**Figure 2.**
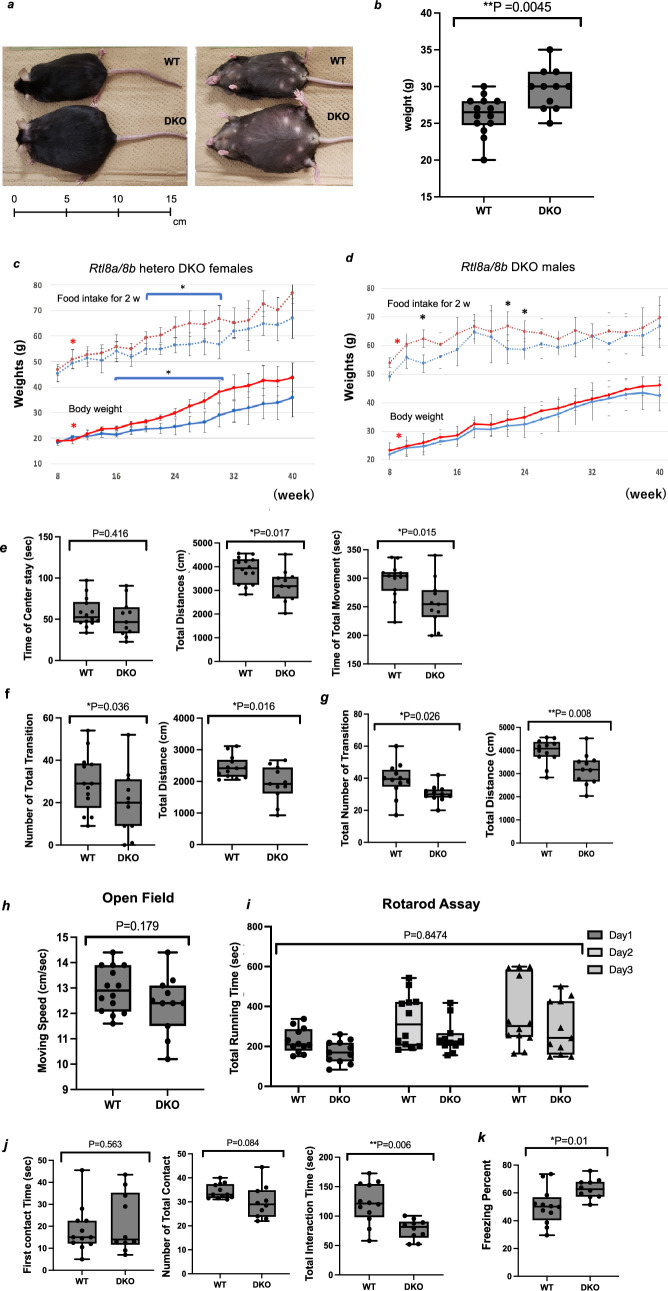
Obesity and abnormal behaviours of *Rtl8a* and *8b* DKO. (*a*) One example of the maximal weight of *Rtl8a* and *8b* hetero DKO females (bottom, 75 g) compared with a WT littermate female (top, 34 g) at 80 w. (*b*) Weight of hemizygous DKO males (*n* = 11) and littermate males (*n* = 14) at 12 w. (*c,d*) Body weight (bottom) and food intake every 2 weeks (top) of the WT (*n* = 5) are shown. Hetero DKO female (*n* = 5) mice and littermate WT females (*n* = 5) (*c*) and hemizygous DKO males (*n* = 5) and littermate WT males (*n* = 5) (*d*). Blue: WT; red: DKO. *p < 0.05. Red asterisks indicate overeating which was observed before the onset of obesity in males and females. (*e–k*) Behaviour tests. Reduced locomotive activity was observed in the OF test (*e*) (WT: *n* = 12, DKO: *n* = 10, *t*‐test), L/D transmission test (*f*) (WT: *n* = 14, DKO: *n* = 11, *t*‐test) and EPM test (*g*) (WT: *n* = 12, DKO: *n* = 11, *t*‐test). *p < 0.05, **p < 0.01. Normal physical capability was observed in the OF test (*h*) (WT: *n* = 14, DKO: *n* = 11, *t*‐test) and rotarod assay (*i*) (WT: *n* = 12, DKO: *n* = 11, two-way ANOVA 0.8474). Reduced social activity was observed in the social behaviour test (*j*) (WT: *n* = 12, DKO: *n* = 10, *t*‐test). Increased apathy-like behaviour was observed in the tail suspension test (*k*) (WT: *n* = 12, DKO: *n* = 10, *t*‐test). *p < 0.05, **p < 0.01.

*Rtl8a*/*Rtl8b* heterozygous (hetero) DKO female mice exhibited abnormal maternal care, and both hetero DKO females and hemizygous DKO males (hereafter referred to as DKO males) (electronic supplementary material, figure S7) exhibited overgrowth from the young adult stage (figure 2). Unexpectedly, half of the hetero DKO females had delivery problems: most of their pups died shortly after birth, regardless of genotype (electronic supplementary material, figure S8, left). In the case of homozygous (homo) DKO mothers, no pups survived (electronic supplementary material, figure S8, right). However, no foetal and postnatal lethality was observed in the DKO pups when their fertilized eggs were transferred into pseudopregnant ICR females and cared for by the ICR mothers. These results suggest abnormal maternal behaviour of the hetero and homo DKO mothers. It is likely that the hetero DKO females may exhibit either normal or abnormal maternal behaviour due to random X chromosome inactivation (XCI) in female mice [45,46].

Both the hetero DKO females and DKO males gradually became obese after 8 weeks of age (w) in association with the increased food intake and this trend continued thereafter (figure 2*a*–*f*). For example, one of the maximum weights of the hetero DKO females reached 75 g at 80 w, compared with the 34 g of a WT littermate female (figure 2*a*) and average weights of hemizygous males and WT littermate males are 29.4 g and 26.1 g at 12 w (figure 2*b*). Interestingly, they exhibited significant overeating (increased food intake/weight ratio) at 9−10 w (hetero DKO females) and at 8−9 w (DKO males), just before the onset of obesity (indicated by red asterisks in figure 2*c*,*d*; see details in electronic supplementary material, figures S9 and S10). These findings suggest some dysfunction of the hypothalamus, a key region in appetite control.

### 2.3. Abnormal behaviours in *Rtl8a/8b* double knockout mice

The DKO mice exhibited reduced locomotive activity, decreased social responses and increased apathy. In the open field (OF: Figure 2*e*, 8 w), light/dark transition (L/D: figure 2*f*, 8 w) and elevated plus-maze tests (EPM: Figure 2*g*, 9 w), the DKO males exhibited reduced locomotive activity in terms of the total distance (3953 versus 3191 cm in OP; *t*‐test 0.017, 2373 versus 1925 cm; *t*‐test 0.016 in L/D and 1961 versus 1653 cm; *t*‐test 0.023 in EPM), total movement (296 versus 257 s; *t*‐test 0.015 in OF) and number of transitions (29.7 versus 17.9 times; *t*‐test 0.036 in L/D and 37.5 versus 30.4 times; *t*‐test 0.046 in EPM). The other elements of these tests were normal (electronic supplementary material, figures S11 and S12) including normal moving speed (OF: figure 2*h*) and a normal score on the rotarod test (Figure 2*i*, 10 w). Although the average scores of the DKO were lower than those in the WT, they are not statistically significant, suggesting their normal physical functioning.

In the social activity test (9 w), the DKO males had similar first contact time and the number of total contacts compared with their WT littermates, but the total interaction time was reduced (121.5 versus 81.6 s; *t*‐test 0.006), suggesting a reduction in social activity (figure 2*j*). In the tail suspension test (Figure 2*k*, 11 w), their freezing time was increased (50.3 versus 63 s; *t*‐test 0.01), suggesting increased apathy. We did not observe any abnormality in the fear conditioning test (12 w) except for lower locomotive activity (electronic supplementary material, figure S13). We also found that some DKO males were severely injured by attacks from other DKO males, while certain other DKO males suffered severe self-inflicted injuries by biting and scratching themselves during normal breeding. These findings suggest that the loss of RTL8A/8B somehow affects neuronal functions in the brain, possibly in the cerebral cortex (see later sections).

### 2.4. mRTL8A/8B protein expression in the brain

**Figure 3.**
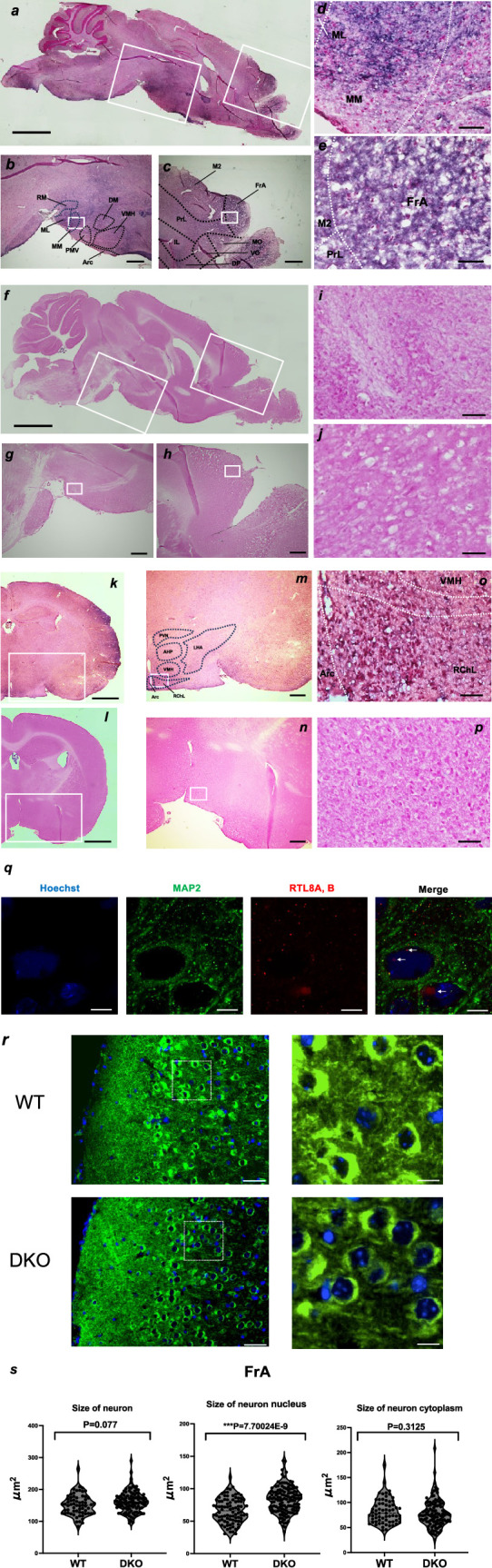
Immunostaining of mRTL8A and 8B proteins in the brain. (*a*) A sagittal section image of the WT mice at 20 w. mRTL8A/8B protein was detected in the frontal cortex and hypothalamus. Scale bar, 1 mm. (*b*) An enlarged hypothalamus area surrounded by the central white square in (*a*). Arc: arcuate nucleus, DMH: dorsomedial hypothalamic nuclei, ML: medial mammillary nucleus, MM: medial mammillary nucleus, PMV: premammillary nucleus, ventral part, RM: retromammillary nucleus, VMH: ventromedial hypothalamic nuclei. Scale bar, 200 μm. (*c*) An enlarged frontal cortex area surrounded by the right-hand white square in (*a*). DP: dorsal peduncular cortex, FrA: frontal association, IL: infralimbic cortex, M2: secondary motor cortex, MO: medial orbital cortex, PrL: prelimbic cortex, VO: ventral orbital cortex. Scale bar, 200 μm. (*d*) A magnified view of the white square in (*b*). Scale bar, 50 μm. (*e*) A magnified view of the white square in (*c*). Scale bar, 50 μm. (*f–j*) Sagittal section images of the DKO mice at 20 w corresponding to (*a–e*). (*k* and *l*) Coronal section images of the WT and DKO at 20 w. Bar: 1 mm. (*m,n*) An enlarged area surrounded by the white square in (*k,j*), respectively. AHP: anterior hypothalamus nucleus, LHA: lateral hypothalamic area, PVN: paraventricular nucleus, RChL: retrochiasmatic area, lateral part. Scale bar, 200 μm. (*o,p*) A magnified view of the white square in (*l,m*), respectively. Scale bar, 50 μm. (*q*) Co-immunofluorescence staining images of MAP2 and RTL8A/8B in the FrA region in WT (sagittal sections, each at 20 w, *n* = 2). Arrows indicate RTL8A/8B in the nuclei. Scale bars, 5 μm. Blue: Hoechst, green: MAP2, red: RTL8A,B. (*r*) Immunofluorescence staining of FrA in WT and DKO mice. Left: immunofluorescence staining images of MAP2 in the FrA region in WT and DKO mice (sagittal sections, each at 20 w, *n* = 2). Right: enlarged images of the dashed box areas in the left-hand images. Green: MAP2, blue: DAPI. Scale bars, 40 μm (left) and 10 μm (right). (*s*) The sizes of the neuronal cells and their nuclei in (*r*) were measured by a blind observer using ImageJ. Three individuals from each of the WT and KO mice were examined for the measurement of a total of 65 and 109 neurons, 88 and 132 neuronal nuclei, and 65 and 109 neuronal cytoplasm samples, respectively (see also electronic supplementary material, figure S17). All measurements were statistically analysed with Student’s *t*‐test and the results are displayed as violin plots. ***p < 0.001.

mRTL8A/8B proteins were detected in certain specific regions, such as the hypothalamus, frontal/temporal cortex, striatum and olfactory bulb (figure 3*a*–*p*; electronic supplementary material, figure S14), which appear to be associated with the abnormal DKO phenotypes mentioned above. There were no significant differences in brain or cortex weight between DKO and WT at 20 w (electronic supplementary material, figure S15). For the detection of the mRTL8A/8B proteins, we used an in-house made anti-mRTL8A/8B antibody by immunization with their unique N-terminal peptides (figure 1*b*; electronic supplementary material, figure S2). We confirmed the antibody specificity by checking whether the WT signals disappeared in the DKO brain (figure 3*f*–*j*,*l*,*n*,*p*).

mRTL8A/8B were detected in a wide area of the prefrontal cortex (PFC), including the orbital cortex, prelimbic cortex (PrL) and frontal association (FrA) (figure 3*c*,*d*), which may be related to abnormal behaviours in DKO mice. The orbitofrontal cortex (OFC) is a ventral subregion of the PFC [47], and involved in the integration of sensory information, emotion processing, decision-making and behavioural flexibility [48,49]. The PrL is involved in depression-like despair behaviour [50] and maintaining attention to category-relevant information, and flexibly updates category representations [51], while the FrA is engaged in stimulus integration during associative learning [52]. The FrA receives projection from the insular (IC) and perirhinal (PRh) cortices in the temporal lobe where high mRTL8A/8B expression was also detected (electronic supplementary material, figure S14). Functional impairment of all these regions may explain the reduced locomotive activity and social interaction as well as the increased apathy-like behaviour of the DKO mice.

mRTL8A/8B were also detected in the most hypothalamic regions (figure 3*a*,*b*,*d*,*k*,*m*,*o*), including several important hypothalamic nuclei related to feeding behaviour, such as the arcuate nucleus (Arc) [53], dorsomedial and ventromedial hypothalamic nuclei (DMH and VMH) [54], paraventricular nucleus (PVN) [55], anterior hypothalamic area, posterior part (AHP) and lateral hypothalamus (LHA) (figure 3*e*,*f*). In addition, they were detected in the nucleus accumbens (Acb) in the ventral striatum (electronic supplementary material, figure S14) and medial forebrain bundle integrated in the LHA that connects the Acb to the ventral tegmental area (VTA) in the midbrain, two important control centres for feeding behaviour [56].

### 2.5. Neuronal expression of RTL8A/8B

Co-immunostaining experiment of mRTL8A/8B with a neuronal marker, microtubule-associated protein (MAP) 2 in the prefrontal cortex, demonstrated that these proteins are localized in both the nucleus (indicated by arrows) and cytoplasm of neurons (figure 3*q*). It is consistent with previous reports that human RTL8A-C protein is expressed in the neurons differentiated from AS and ALS patient iPSCs (iNeurons) [22,23] and also with the fact that human RTL8A/8B are involved in the nuclear transition of UBQLN2 that is necessary for nuclear protein quality control [29]. It is also consistent with our results that mRTL8A/8B with the N-terminal NLS-like signal is accumulated in the nuclei of NIH3T3 cells while mRTL8C without it is located in the cytoplasm (figure 1*c*).

Immunofluorescence staining with anti-MAP2 antibody also demonstrated that the nuclei of the layer 2/3 pyramidal cells in the FrA were significantly increased in size (figure 3*r*,*s*; electronic supplementary material, figures S16 and S17) in the 20 w adult brain. This was not observed in the other cortical regions without mRTL8A/8B expression, such as the primary somatosensory cortex, shoulder region (S1Sh) (electronic supplementary material, figures S18–S21). It has been reported that the pyramidal cells in the mPFC moderate stress-induced depressive behaviours [57], suggesting that this neuronal alteration is related to the apathy-like behaviour observed in the DKO mice possibly due to the abnormal nuclear protein quality described above. We did not observe any signs of neuroinflammation by immunofluorescence staining for Ubiquitin and glial fibrillary acidic protein (GFAP) (electronic supplementary material, figure S22).

### 2.6. Reduced expression of GABRB2 in the double knockout cerebral cortex

**Figure 4.**
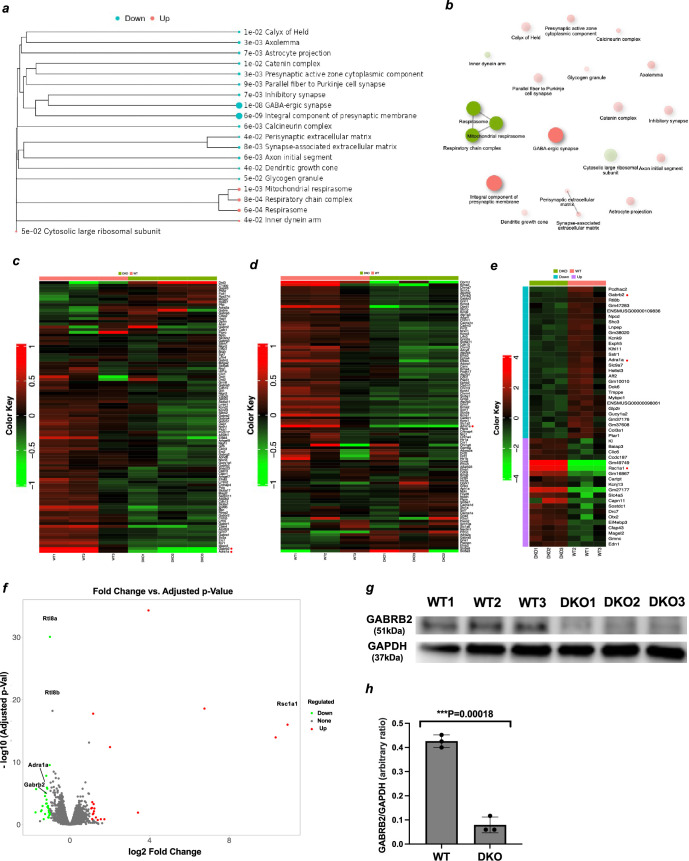
Differentially expressed genes between DKO and WT cerebral cortex. (*a*) The tree plot shows the result of gene set enrichment analysis using the GO cellular component terms (FDR < 0.05). Light blue and pink represent downregulated and upregulated genes, respectively. Circle size correlates with the number of genes in each gene set. (*b*) The network diagram shows the relationship between the results. Two pathways (nodes) are connected if they share 30% (default, adjustable) or more genes. Green and red represent up- and downregulated pathways, respectively. Darker nodes represent more significantly enriched gene sets. Larger nodes represent larger gene sets. Thicker edges represent more overlapping genes. (*c,d*) The heat maps show the expression level (normalized fold change (FC) of each individual for the gene expression level categorized by ‘integral component of presynaptic membrane’ (*c*) and ‘GABAergic synapse’ (*d*)). Red dots indicate genes suspected to be associated with the DKO phenotype. Left panel colour indicates log_2_FC. (*e*) The heatmap of a total of 20 upregulated and 28 downregulated genes that exhibited twofold increase and decrease, respectively. Red dots indicate genes suspected to be associated with the DKO phenotype. (*f*) The volcano plot shows the FC and the FDR of the genes analysed in (*a*). The names of the genes that appear to be particularly associated with the DKO phenotype are indicated. X = FC, Y = −log_10_(FDR). (*g*) The results of western blotting using antibodies against GABRB2 and GAPDH. (*h*) The results of (*g*) quantifying the expression levels using imageJ’s Gel tool.

RNAseq analysis suggested that *Gabrb2*, the β2 subunit of the GABA_A_ receptor known to be an important psychiatric factor, was downregulated in the DKO cerebral cortex, and this was confirmed at the protein level by western blotting. To elucidate the cause of the abnormal behaviour in DKO, we performed RNAseq analysis on the 20 w cerebral cortex of DKO and WT. Importantly, the gene set enrichment analysis (GSEA) using the GO cellular component terms (false discovery rate (FDR) < 0.05) revealed that the top two most downregulated gene sets were ‘integral component of presynaptic membrane’ (6e−09) and ‘GABAergic synapse’ (1e−08) (figure 4*a*–*d*), and only three GO terms, ‘respirasome’, ‘respiratory chain complex’ and ‘mitochondrial respirasome’, were significantly upregulated (figure 4*a*,*b*; electronic supplementary material, figures S23–S25). However, among them, only *Gabrb2* and *Adra1a* remained as downregulated genes under the condition of twofold increase and decrease in a total of 20 upregulated and 28 downregulated genes (figure 4*e*,*f*).

Both *GABRB2* and *ADRA1A* have been reported to be associated with several psychiatric disorders: the former with SZ, BP, FTD and ASD [34–42] and the latter with attention deficit hyperactivity disorder (ADHD), SZ and generalized anxiety disorder (GAD) [58–60], thus making them good candidates for the DKO phenotypes. We hypothesized that the downregulation of *Gabrb2* in the cerebral cortex is a better candidate to explain the abnormalities in DKO mice, as *GABRB2* has been reported to be decreased in the prefrontal cortex of elderly depressed patients [61]. We, then, performed western blot analysis of GABRB2 and confirmed a fivefold reduction in GABRB2 protein expression in the cerebral cortex of 1-year-old DKO mice (figure 4*g*,*h*).

## 3. Discussion

### 3.1. Roles of eutherian-specific RTL8A, 8B and 8C in the brain

This study demonstrates that the retrovirus-derived *Rtl8a* and *8b* genes, which encode proteins with only 113 aa, play important roles in the brain through their involvement in the regulation of certain basic behaviours, such as social activity, emotions, maternal care and food intake. This explains why *RTL8A-C* is well conserved in eutherians (electronic supplementary material, table S1). Given that there is a gene dosage effect for *RTL8A-C*, this may also explain why *RTL8A-C* exists as a cluster of multiple genes, for example, a kind of triple safety valve in the eutherian brain. In this work, mouse *Rtl8a* and *Rtl8b* were knocked out; however, *Rtl8c* still remained active, so it is of interest to determine whether complete null mice, i.e. *Rtl8a*, *Rtl8b* and *Rtl8c* triple KO (TKO) mice, will exhibit more severe phenotypes and more severe gene expression changes than the DKO mice, as discussed below. A comparison between *Rtl8c* KO, *Rtl8a* and *Rtl8b* DKO and *Rtl8a*, *Rtl8b* and *Rtl8c* TKO mice would also provide important insights into the role of *Rtl8a*, *Rtl8b* and *Rtl8c* in both the cerebral cortex and hypothalamus, the molecular mechanisms of these genes in these abnormalities and the reason why these three genes are evolutionarily conserved in eutherians.

### 3.2. Down- and upregulated genes in *Rtl8a and Rtl8b* double knockout cerebral cortex

As shown in figure 4, *Gabrb2* and *Adra1a* are nominated as good candidate genes responsible for DKO psychiatric abnormalities in the 20 w cerebral cortex as downregulated genes. Genome-wide association studies (GWAS) reveal that common variants within *GABRB2* are associated with an increased risk of SZ [34–38], BD [39], FTD [40] and ASD [36]. In addition, it has been reported that *GABRB2* is decreased in the prefrontal cortex of elderly depressed patients [61]. Therefore, it is likely that the downregulation of GABRB2 in the cerebral cortex (figure 4*g*,*h*) at least partially causes the reduced social activity and increased apathy in the DKO mice, although the mechanism by which the absence of *Rtl8a* and *8b* downregulates the *Gabrb2* expression requires further investigation. The DKO exhibits a similar phenotype of sociability impairment of *Gabrb2* KO mice [62] but with some differences, such as a schizophrenia-like phenotype, particularly increased body weight and food intake [62]. It is likely that the differences are due to the fact that *Gabrb2* KO mice lack GABRB2 expression throughout the brain and that the phenotypes of our DKO mice are more complex because many other candidate genes besides *Gabrb2* are also affected with respect to cerebral cortex function and have other causes for the defects derived from abnormalities in the hypothalamus.

As *ADRA1A* has also been reported to be associated with ADHD, SZ and generalized anxiety disorder by GWAS [58–60], a detailed study of *Adra1a* with respect to its contribution to the DKO phenotypes is warranted in the future. It should be noted that Regulator of Solute Carriers 1 (*Rsc1a1*) exhibited an exceptional overexpression (approximately 12-fold) in DKO (figure 4*e*,*f*). It is possible that the *Rsc1a1* overexpression somehow compensates for the absence of RTL8A/8B. RSC1A1 is located on the intracellular side of the plasma membrane, at the trans-Golgi network and in the nucleus [63]. Therefore, it is interesting to know whether RTL8A/8B and RSC1A1 proteins colocalize and functionally cooperate in the brain. RSC1A1 possesses the consensus sequences for the ubiquitin-associated (UBA) domain in addition to those for phosphorylation by PKC and casein kinase 2 [64]. UBQLN2 also possesses the UBA domain, which is required for incorporation of UBQLN2 into RTL8 subnuclear puncta, although it is not required for direct binding to RTL8 [29]. Therefore, it could also be possible that *Rsc1a1* overexpression somehow disrupts UBQLN2 function, such as trafficking of ubiquitinated proteins to proteasomes, via competition between their UBA domains. As shown in figure 4*a*,*b*, genes categorized as ‘respirasome’, ‘respiratory chain complex’ and ‘mitochondrial respirasome’ were also slightly upregulated (electronic supplementary material, figures S23–S25). These upregulated genes will be further investigated in the TKO mice in addition to *Rsc1a1*.

### 3.3. Possible link of *RTL8A*/*8B* to human diseases

The involvement of RTL8 has been implicated in two human diseases, ALS [23] and AS [22], where RTL8 decreases in the former and accumulates in the latter. The situation in DKO is similar to the former; however, the DKO mice had a normal score in the rotarod test (figure 2*k*), suggesting that downregulation of these genes is not correlated with the ALS phenotype [23], although a TKO study will be required for a definitive conclusion. It is also of interest whether overexpression of human RTL8A-C together with overexpression of PEG10 (aka *RTL2*), another RTL gene, is involved in the aetiology of AS [22]. However, this will require another mouse model overexpressing *Rtl8a-c*.

It should be noted that the DKO phenotypes, the overgrowth due to increased food intake after 8 w (figure 2*a*–*f*), as well as reduced social interaction (figure 2*l*) and increased apathy (figure 2*m*), are quite similar to those observed in the late PWS patients, who present with late-onset obesity and ASD-like symptoms from the juvenile age [24,25]. By contrast to AS, which is caused by a paternal duplication of the chromosome 15q11-q13 and/or a UBE3A mutation [24,26,27], PWS, another neurodevelopmental genomic imprinting disorder, is caused by its maternal duplication, resulting in overexpression of UBE3A. Thus, it is probable that RTL8A-C is reduced in PWS patients, as it is in the DKO mice. It is then of interest to investigate whether the RTL8A-C protein is reduced in the PWS brain. To understand the aetiology of late-onset obesity, further investigation of gene expression changes in the DKO hypothalamus is needed. It should be noted that in addition to the functional impairment of the hypothalamic regions, the medial orbital cortex (MO), a part of the orbitofrontal cortex (OFC), may explain the late-onset obesity, since the MO has been reported to play an important role in the regulation of feeding behaviour [65].

RTL8A-C is located on Xq26, far from the causative PWS chromosomal region (15q11-q13). Interestingly, however, a de novo paracentric inversion (X)(q26q28) with features mimicking PWS has been reported [66], suggesting the possibility that RTL8A-C may be responsible for the late PWS phenotypes. GABA_A_ receptor alterations have been reported in PWS patients, although it remains to be determined which α and β subunits are affected, i.e. α5 and β3 on chromosome 15q11-q13 (the PWS region) or α1 and β2 on chromosome 5q34-q35 or both [67]. It will then be interesting to know whether *GABRB2* expression is actually downregulated in PWS patients.

In addition to *RTL8A-C*, *RTL1* and *RTL4* have been implicated in neurodevelopmental disorders, such as Kagami-ogata and Temple syndromes [13,17,18,68–71] and ASD [15,16]. Eutherian-specific retroviral *Gag*-derived genes should therefore be the focus of more attention as important targets in human neurodevelopmental disorders.

## 4. Material and methods

### 4.1. Animals

All of the animal experiments were reviewed and approved by the Institutional Animal Care and Use Committee of RIKEN Kobe Branch, Tokai University and Tokyo Medical and Dental University (TMDU) and were performed in accordance with the RIKEN Guiding Principles for the Care and Use of Laboratory Animals, and the Guideline for the Care and Use of Laboratory Animals of Tokai University and TMDU.

### 4.2. Estimation of the pairwise dN/dS ratio

Conversion of the protein sequence alignment created with the MAFFT program (https://mafft.cbrc.jp/alignment/server/index.html) into the corresponding codon alignment was performed with the PAL2NAL program (https://www.bork.embl.de/pal2nal/) [72]. The PAL2NAL program simultaneously calculated the nonsynonymous/synonymous substitution rate ratio (dN/dS) by the CodeML program (runmode: −2) in PAML [73]. An aa sequence phylogenic tree was constructed with MEGA5 using the maximum likelihood method based on the JTT matrix based model [74]. The bootstrap consensus tree inferred from 1000 replicates is taken to represent the evolutionary history of the taxa analysed. Branches corresponding to partitions reproduced in less than 50% bootstrap replicates are collapsed. The percentages of replicate trees in which the associated taxa were clustered together in the bootstrap test (1000 replicates) are shown next to the branches. Initial tree(s) for the heuristic search were obtained automatically as follows. When the number of common sites was <100 or less than one fourth of the total number of sites, the maximum parsimony method was used; otherwise the BIONJ method with MCL distance matrix was used. The tree is drawn to scale, with branch lengths measured in terms of the number of substitutions per site. The analysis involved 10 aa sequences. All positions containing gaps and missing data were eliminated. There was a total of 104 positions in the final dataset.

The *RTL8A*, *8B* and *8C* genome sequences used for the dN/dS analysis (electronic supplementary material, table S1) were as follows. Human: NC_000023.11[c135052108-135051767, c135022459-135022118, 135032384-135032725]; Chimpanzee: NC_036902.1[c130245552-130245211, c130216735-130216394, 130226101-130226442]; Marmoset: NC_013918.1[122938486-122938827, c122959788-122959447, c123007085-123006744]; Mouse: NC_000086.8[c52645588-52645247, c52672265-52671924, 52610013-5 26 10 351]; Cow: NC_037357.1[18783020-18783361, 18794515-18794856, 18824715-18825056]; Rabbit: NC_013690.1[109509895-109510236, 109553752-109554093, 109580009-10958035]; Dog: NC_049780.1 [106817683-106818024, 106834142-106834483, 106843724-106844065, 106859125-106859466]; Elephant: NW_003573520.1[4927338-4927679, c 4938179-4937838] and NW_003575145.1[c434-87].

### 4.3. Amino acid identity and similarity

Amino acid identity and similarity were calculated using the EMBOSS Water program (http://www.ebi.ac.uk/Tools/psa/emboss_water/) in the default mode.

### 4.4. Subcellular localization of the mRTL8A and 8C in NIH3T3 cells

For overexpression of mRTL8A and 8C proteins, the ORF (open reading frame) of *mRtl8a* and *mRtl8c* together with the Kozak sequence were amplified by PCR and cloned into pcDNA-3.1-myc His (Invitrogen) using NheI and ApaI restriction enzymes. The sequences were confirmed by sequencing. PCR was performed under the following conditions using the specified primers: reaction mixture (total 25 µl) contained 12.5 µl of 2× KOD FX buffer, 5 µl of dNTP mix (2.0 mM each), 0.04 µl of forward and reverse primers (200 pmol µl^−1^ each), 1.0 µl of genomic DNA (5 ng µl^−1^), 0.5 µl of KOD FX polymerase (TOYOBO) and 6.42 µl of DDW, and the PCR condition was 1 cycle at 96°C for 45 s, followed by 35 cycles of 96°C for 15 s, 61°C for 30 s, and 72°C for 30 s. The following primers were used for *Rtl8a*, forward: 5′-gctagcgccatggaaggccaaggcaaggtaaaga-3′ and reverse: 5′-gggccccgggtgggaggaggatgaggacttc-3′ and for *Rtl8c*, forward: 5′-gctagcgccatggaccgccggattaagttgatta-3′ and reverse: 5′-gggcccgaagtcctcatcctcctcccacccg-3′.

Transfection of the plasmid into NIH3T3 cells was performed using polyethyleneimine (PEI) MAX (Polysciences, Inc.). Cell culture and transfection conditions were as follows: NIH3T3 cells were cultured in DMEM (Dulbecco’s modified Eagle medium) supplemented with 10% FBS (foetal bovine serum) and 0.1% penicillin/streptomycin until reaching 70% confluence on the day before transfection. Prior to transfection, the cells were washed with PBS and then incubated with 350 µl of 10% FBS DMEM. The transfection mixture containing 1.16 µl of 1 mg ml^−1^ PEI MAX, 386 ng of plasmid DNA and 35 µl of 1.5 M NaCl was added to the cells. After 24 h of incubation, the cells were used for western blot and immunostaining. The cells were washed with PBS, fixed with 4% paraformaldehyde, permeabilized with 0.1% Triton-X100 and blocked with 10% goat serum albumin, then incubated with primary antibody followed by secondary antibody conjugated to Alexa Fluor 488. Nuclei were stained with DAPI and images were captured using a confocal microscope.

### 4.5. Production of RTL8A and RTL8C chimeric proteins

The ORF sequences used for RTL8A and 8C overexpression were ligated into pcDNA-3.1-myc His after digestion with PciI restriction enzyme. Chimera constructs were then selected by sequencing. Overexpression and immunostaining were performed using the same methods as for RTL8A and RTL8C overexpression.

### 4.6. RT-PCR

RT-PCR was performed using cDNA. cDNA was synthesized from 1 μg of total RNA using SuperScript III Reverse Transcriptase (Invitrogen). RNA was extracted from adult tissues at 19 weeks, including the cerebrum, cerebellum, heart, liver, lung, spleen, kidney, stomach, intestine, large intestine, skeletal muscle, thymus, bladder, adipose, uterus, ovary, testis, epididymis and vesicular gland as well as day 9.5 placenta by treatment with TRIzol Reagent (Life Technologies). Ten nanograms of cDNA were mixed with 1× ExTaq buffer (TAKARA), 2.5 mM of each dNTP 2 μl, primer 0.2 μl (200 pmol μl^−1^) and ExTaq HS 0.1 μl (TAKARA), and PCR analysis was performed under the following conditions. *Rtl8a*, *b* and *c*: 95℃ 1 min, 32 (*Rtl8a* and *b*) and 31 (*Rtl8c*) cycles at 96℃ for 10 s, 72℃ for 30 s and 72℃ for 90 s. *β-actin*: 95℃ 1 min, 26 cycles at 96℃ for 10 s, 72℃ for 30 s and 72℃ for 90 s using a C1000 Touch thermal cycler (Bio-Rad). The following primer sets were used. *Rtl8a*, *b* F1: tcccactgttgacagctcag and *Rtl8a*, *b* R1: ggggctactgttggaaaa, and *Rtl8c* F1: tatgcgctatctaaagacagac and *Rtl8c* R1: ggcttctgtacaggtagaggcagac. The RFLP (restriction fragment length polymorphism) experiment was carried out using *Msp*I, and the results were confirmed by electrophoresis.

### 4.7. Generation of *Rtl8c* flox mice

A targeting construct spanning the two exons of *Rtl8c* was used to generate *Rtl8c* flox mice (accession no. CDB0788K: https://large.riken.jp/distribution/mutant-list.html). Briefly, the construct included two loxP sites upstream of exon 1 and downstream of exon 2, and a frt-neo-frt cassette downstream of the latter loxP site. After linearizing, the construct was electroporated into TT2 ES cells (C57BL/6 × CBA genetic background) [75]. Two ES clones were then screened by PCR and southern blotting. One 5′ and one 3′ probe, both of which were outside of targeting construct, were used in southern blotting. The correct targeting clones were then used to generate chimera founder lines. Germline transmission was observed in two chimeric mice.

The *Rtl8c* flox alleles were checked by electrophoresis of the PCR amplification using the following primers. WT forward: GGCGACAACCAAGGTTTTTA and reverse: GGGTCTGCTTCTCTTTGCTG, flox allele forward: AAATAGGCGTATCACGAGGC and reverse: GGGTCTGCTTCTCTTTGCTG.

The PCR cycle was 96℃ 1 min, 35 cycles at 96℃ for 15 s, 60℃ for 30 s and 72℃ for 30 s and 72℃ 2 min.

### 4.8. Generation of *Rtl8a/Rtl8b* double knockout mice

The DKO mice were generated with reference to our previous report [76] as follows. The plasmids expressing *hCas9* and sgRNA were prepared by ligating oligos into the BbsI site of pX330 (Addgene plasmid 42230). The 20 bp sgRNA recognition sequence was: *Rtl8a, b*-sgRNA (5′-ACGGGATGGGGTTCCGCCGA-3′). The sgRNA targets both *Rtl8a* and *Rtl8b.*

To produce the *hCas9* mRNA, the T7 promoter was added to the *hCas9* coding region of the pX330 plasmid by PCR amplification, as previously reported [77]. The T7-*Cas9* PCR product was gel purified and employed as the template for *in vitro* transcription (IVT) using a mMESSAGE mMACHINE T7 ULTRA kit (Life Technologies). The T7 promoter was added to the *Rtl8a, b*-sgRNA region of the pX330 plasmid by PCR amplification using the following primers. *Rtl8a, b*-sgRNA-F (5′-TGTAATACGACTCACTATAGGGACGGGATGGGGTTCCGCCGA-3′), *Rtl8a, b*-sgRNA-R (5′-AAAAGCACCGACTCGGTGCC-3′). The T7-sgRNA PCR product was gel-purified and employed as the template for IVT using a MEGAshortscript T7 kit (Life Technologies). Both the *hCas9* mRNA and *Rtl8a, b*-sgRNA were DNase-treated to eliminate template DNA, purified using a MEGAclear kit (Life Technologies), and eluted into RNase-free water.

C57BL/6N female mice were superovulated and *in vitro* fertilization was carried out using *Rtl8c* flox mouse sperm. The synthesized *hCas9* mRNA (50 ng μl^−1^) and *Rtl8a, b*-sgRNA (25 ng μl^−1^) with oligo DNA (50 ng μl^−1^) were injected into the cytoplasm of fertilized eggs at the indicated concentration. The eggs were cultivated in KSOM overnight, then transferred into the oviducts of pseudopregnant ICR females. The oligo DNA designed as the HDR (homology-directed repair) donor induced a nonsense mutation in both *Rtl8a* and *Rtl8b* and harboured an accompanying AflII recognition site used for PCR-RFLP (Restriction Fragment Length Polymorphism) genotyping. The sequence of the oligo DNA was: 5′-AAGGCCAAGGCAAGGTAAAGAGGCCGAAGGCCTACATGCTCAGGCACAACAGGCGGCGCCCTTAAGGGAACCCCATCCCGTTTCCAGAGCTGTTTGATGGCGAGATGGACAAGCTCCCGGAGTTCA-3′.

### 4.9. Genotyping of *Rtl8a* and *8b* double knockout mice

The genotype was determined by PCR-RFLP analysis. The following primers were used for PCR amplifications. *Rtl8a*: 5′-GGACTGGCGCCTGAAATAGC-3′ and 5′-GCACAATCACCACCTCTTGAACA-3′, *Rtl8b*: 5′-CCACCCCTTAAACATTCTCCTGG-3′ and 5′-AGATCGAACATCAGGCCATGAAC-3′. The PCR products were digested with AflII and subjected to agarose gel electrophoresis.

### 4.10. Behavioural analysis

Behavioural analysis was performed as previously described [17,19]. Briefly, both DKO and littermate WT male mice (8–13 w) were analysed in the following order: light and dark transition (8 w, WT: *n* = 14, KO: *n* = 11), open field (8 w, WT: *n* = 14, KO: *n* = 11), social interaction (9 w, WT: *n* = 12, KO: *n* = 10), elevated plus maze (9 w, WT: *n* = 12, KO: *n* = 11), rotarod test (10 w, WT: *n* = 12, KO: *n* = 11), tail suspension (11 w, WT: *n* = 12, KO: *n* = 11) and fear condition tests (12 w, WT: *n* = 12, KO: *n* = 11).

#### 4.10.1. Light and dark transition test

The apparatus used for the light/dark transition test comprised a cage (21 × 42 × 25 cm^3^) divided into two sections of equal size by a partition with a door (O’hara & Co. Ltd). One chamber was brightly illuminated (400 lux), whereas the other chamber was dark. Each mouse was placed into the dark side and allowed to move freely between the two chambers with the door open for 10 min. The total number of transitions between the chambers, the time spent in each, the latency to first enter the light chamber, and the distance travelled in each chamber were recorded using a video camera attached to a computer and calculated by the Image LD program.

#### 4.10.2. Open field test

Locomotor activity was measured using an open field test. Each mouse started the test in the corner of the open field apparatus (40 × 40 × 30 cm^3^; Accuscan Instruments, O’hara & Co. Ltd). The chamber of the test was illuminated at 100 lux. Total distance (cm), total move time and time spent in the centre area were recorded.

#### 4.10.3. Social interaction test

Sociability was measured using the open field apparatus. Mice of the same genotype were placed at diagonal corners in an open field apparatus in small baskets. After waiting for 1 min, the behaviour was recorded for 10 min after the mice exited from the baskets at the same time. The time to first contact, the total number of contacts and the total time of sociable behaviour were analysed. All data were analysed by a blind observer. The total sociable behaviour time was measured excluding contact of less than 1 s. The test was performed with different pairs for a period of 2 days, and the average value for the 2 days was calculated. Mice were paired such that the weight difference was 2 g or less.

#### 4.10.4. Elevated plus maze test

The elevated plus maze consisted of two open arms (25 × 5 cm^2^) and two closed arms of the same size with 15 cm high transparent walls (O’hara & Co. Ltd). The behaviour testing room (170 × 210 × 200 cm^3^) was soundproof, and the illumination level was maintained at 100 lux. Each mouse was placed in the central square of the maze with its head pointed towards a closed arm. Data were recorded for 10 min using a video camera attached to a computer and were calculated with the Image EP program. The number of entries into each arm, total arm entries and the time spent in the open arms were recorded.

#### 4.10.5. Rotarod test

Balance and motor ability were confirmed by the rotarod (BrainScience.idea.co. Ltd). After placing the mouse in the rotating lane, the speed was changed from 3 to 30 r.p.m. and the time until the mouse fell was measured. When one half of the body of the mouse was detached from the rods, it was measured as if it had fallen. The test was conducted for 3 consecutive days and 3 times a day.

#### 4.10.6. Tail suspension test

Depression was investigated using a tail suspension test. The device used was the same as the fear test described next. The mouse was hung on a hook with tape, and observed for 10 min. Recordings were made with a camera attached to the device and the freezing percentage was measured with software (O’hara & Co. Ltd). Freezing percentages were compared during the time elapsed excluding the first 3 min.

#### 4.10.7. Fear condition test

On the first day, each mouse was placed into a test chamber (26 × 34 × 29 cm^3^) with a stainless‐steel grid floor inside a sound‐attenuated chamber and allowed to explore freely for 120 s. A 60 dB white noise acting as a conditioned stimulus (CS) was presented for 30 s, followed by a mild foot shock (2 s, 0.5 mA) acting as an unconditioned stimulus (US). Two more CS–US pairings were given at a stimulus interval of 2 min. On the second day, a context test was performed in the same chamber as the conditioning test and data were recorded for 2 min. On the third day, a cued test was performed in another testing chamber. The same 60 dB white noise as on the first day was given for 30 s and data were recorded for 2 min. The entire test was recorded by a video camera attached to a computer. In each test, the movement freezing percentage and total move distance were calculated automatically by the Image FZ program.

### 4.11. Immunohistochemistry

Mouse adult brains were fixed in 4% paraformaldehyde (PFA: Nacalai tesque), incubated in 10% and 25% sucrose at 4℃ overnight each and finally embedded in OCT compound (Sakura Finetek). The OCT blocks were sectioned at a 13 μm thickness with a cryostat (MICROTOME) and mounted on Superfrost Micro Slides (Matsunami Glass). The cryosections were fixed in 4% PFA for 10 min at room temperature and washed three times with PBS for 5 min. For antigen retrieval, the sections were boiled in 0.01 M citrate buffer (pH 4.0) at 98℃ for 30 min (for RTL8A/8B), and then immersed (dehydrated) in cold methanol at −30℃ for 30 min. After being air dried, the sections were blocked with 10% goat serum, 1% bovine serum albumin (BSA: Sigma Aldrich) and 0.1% Triton‐X100 (WAKO) in PBS at room temperature for 1 h.

For the staining, an anti‐RTL8A/8B antibody (SCRUM 1:1000) was used as the primary antibodies and were prepared in 1% BSA and 0.1% Triton‐X100 in PBS at 4℃ overnight. The second antibody was Biotin-αRabbit-IgG (VECTOR STAIN 1:200). Nuclei were stained with nuclear fast red. The slides were mounted with malinol (MUTO Pure Chemicals, Tokyo, Japan). The images were captured using a BIOREVO microscope (KEYENCE). Three WT and three DKO mice were used for immunohistochemisry.

### 4.12. Immunofluorescence staining using floating frozen section

Mouse brains were fixed and embedded in OCT blocks as described in §4.11. The OCT blocks were thinly sliced at 50 µm, floated in a glass-bottom dish with 2 ml of PBS and washed for 10 min. PBS was removed and 2 ml of 0.1 M PBS/0.3% Triton was added and washed for 15 min three times. The sections were blocked with 10% donkey serum, 5% BSA (Sigma Aldrich) and 0.1% Triton‐X100 (WAKO) in PBS at room temperature for 1 h. For the staining, an anti‐RTL8A/8B (SCRUM 1:2000) and anti-MAP2 (ab5392 1:20 000) antibodies were used as the primary antibodies and were prepared in 5% BSA and 0.1% Triton‐X100 in PBS at 4℃ overnight. Second antibodies used were the Alexa Fluor 488 donkey anti-Mouse IgG (H+L) (Life technologies, A21203, 1:2000) and Alexa Fluor 488 donkey anti-Rabbit IgG (H+L) (Jackson ImmunoResearch, 711-545-152, 1:2000) and were prepared in 5% BSA and 0.1% Triton‐X100 in PBS at 4℃ overnight. Nuclei were stained with DAPI (VECTOR STAIN, 1:1000). The slides were mounted with VECTERSHIED Hardset antifade mounting medium (VECTOR STAIN). The images were captured using a confocal laser microscope (TCS SP8, Leica). Three WT and three DKO mice were used for immunostaining.

### 4.13. Immunofluorescence staining using paraffin-embedded samples

Mouse adult brains were fixed overnight using 4% PFA (Nacalai tesque), and soaked in 70% ethanol at 4℃ overnight, then dehydrated in 70%, 80%, 90% ethanol for 1 h each, 100% ethanol for 2 h twice and 3 h once. They were embedded in paraffin wax after treatment with Gnox (Genostaff, GN04) for 2 h twice, Gnox and paraffin for 2 h, and paraffin for 3 h and 4.5 h. The paraffin blocks were sectioned at a 5 μm thickness with a microtome and mounted on Superfrost Micro Slides (Matsunami Glass). For antigen retrieval, the sections were boiled in 0.01 M citrate buffer (pH 6.0) at 90℃ for 15 min then immersed (dehydrated) in cold methanol at −30℃ for 30 min. After being air dried, the sections were treated with 10% goat serum, 1% BSA (Sigma Aldrich) and 0.1% Triton-X100 (WAKO) in PBS at room temperature for 1 h.

For the immunofluorescence staining, anti-MAP2 (Proteintech 17490-1-AP 1:200) antibody was reacted as the primary antibodies in 1% BSA and 0.1% Triton-X100 in PBS at 4℃ overnight (approximately 16 h). Second antibodies used were the Alexa Fluor 488 donkey anti-Rabbit IgG (H+L) (Jackson ImmunoResearch, 711-545-152, 1:1000). Nuclei were stained with DAPI (VECTOR STAIN, 1:1000). The slides were mounted with VECTERSHIED Hardset antifade mounting medium (VECTOR STAIN). Neuronal areas were measured by a blind observer using ImageJ. The neuronal cells and nuclei were outlined manually with ‘Polygon selections’ tool. The area of MAP2-positive neurons and the area of the nucleus were measured. Cytoplasmic area was calculated by subtracting nuclear area from cell area. Three WT and three DKO mice were used for immunostaining.

### 4.14. RNA-Seq

For RNA isolation and quality control, the integrity and quantity of the total RNA was measured using an Agilent Technologies 2200 TapeStation (Agilent Technologies Inc., Santa Clara, CA). For library preparation and sequencing, total RNA obtained from each sample was subjected to a sequencing library construction using the TruSeq Stranded mRNA Library Prep Kit (Illumina Inc., San Diego, CA) according to the manufacturer’s protocols. Sample libraries were sequenced using NovaSeq 6000 (Illumina Inc., San Diego, CA) in 100 base pair (bp) paired-end reads. For alignment to the whole transcriptome, quantification of gene expression levels and detection of differentially expressed genes, sequencing adaptors, low-quality reads and bases were trimmed using the Trimmomatic-0.39 tool [78]. Sequence reads were aligned to the mouse reference genome (mm10) using STAR 2.7.9 a [79]. The aligned reads were subjected to downstream analyses using StrandNGS 4.0 software (Agilent Technologies Inc.). Read counts assigned to each gene and transcript (Ensembl Database 2018.02.25) were quantified using an iDEP 1.12 [80] with the DESeq2 package. Finally, the gene set enrichment analysis was performed using this platform with GO cellular component gene sets (FDR < 0.05). The tree plot and network diagram were classified according to the GO cellular component process collection. Three WT and three DKO mice were used for RNAseq.

### 4.15. Western blot

Membrane proteins were extracted from mouse brain using a Thermo Scientific Mem-PER Plus Membrane Protein Extraction Kit (Thermo: 89842). The extracted proteins were boiled in 4× sample buffer at 95°C for 3 min. Electrophoresis was then performed on a 4−20% gradient gel (BIO-RAD: 4561095) at 100 V for 40 min. After electrophoresis, the gel was transferred to membrane (BIO-RAD: 1704156) and blocked overnight with 5% skim milk in TBS. Primary antibodies, GABRB2 (Sigma: ZMS1130, 1/1000) and GAPDH (Proteintech: 10494-1-AP, 1/20 000), were incubated with the membrane overnight at 4°C. Secondary antibodies, anti-mouse (Proteintech: SA00001-2, 1/2000) and anti-rabbit (Proteintech: SA00001-2, 1/2000), were then incubated for 2 h at room temperature. The reaction was visualized by adding detection reagent (GE Healthcare: 28980926), and images were captured using a gel documentation system (ImageQuant™ LAS 4010/4000). Protein expression levels were measured using the imageJ gel tool and corrected for GAPDH levels. Three WT and three DKO mice were used for western blot.

## Data Availability

We have deposited the results of RNAseq data on Rtl8a and 8b DKO cerebral cortex. Accession to cite for these SRA data: PRJNA1056858. Electronic supplementary material is available online [81].
